# The secretome of *Thermococcus barophilus* in the presence of carbohydrates and the potential role of the TrmBL4 regulator

**DOI:** 10.1111/1758-2229.13186

**Published:** 2023-07-26

**Authors:** Maria Batour, Sébastien Laurent, Yann Moalic, Hala Chamieh, Samir Taha, Mohamed Jebbar

**Affiliations:** ^1^ Univ Brest, CNRS, Ifremer, Laboratoire de Biologie et d'Écologie des Écosystèmes marins profonds (BEEP), IUEM Plouzané France; ^2^ Laboratory of Applied Biotechnology, Azm Center for Research in Biotechnology and Its Applications Lebanese University Tripoli Lebanon; ^3^ LabISEN, Yncréa Ouest Brest France

## Abstract

Global transcriptional regulators are crucial for supporting rapid adaptive responses in changing environments. In Thermococcales, the TrmB sugar‐sensing regulator family is well represented but knowledge of the functional role/s of each of its members is limited. In this study, we examined the link between TrmBL4 and the degree of protein secretion in different sugar environments in the hyperthermophilic Archaeon *Thermococcus barophilus.* Although the absence of TrmBL4 did not induce any growth defects, proteomics analysis revealed different secretomes depending on the sugar and/or genetic contexts. Notably, 33 secreted proteins present in the supernatant were differentially detected. Some of these proteins are involved in sugar assimilation and transport, such as the protein encoded by *TERMP*_*01455* (cyclomaltodextrin glucanotransferase), whereas others have intracellular functions, such as the protein encoded by *TERMP*_*01556* (pyruvate: ferredoxin oxidoreductase Δsubunit). Then, using reverse transcription quantitative polymerase chain reaction experiments, we observed effective transcription regulation by TrmBL4 of the genes encoding at least two ABC‐type transporters according to sugar availability.

## Graphical abstract

The secretome of Thermococcus barophilus was explored in the light of sugar metabolism (maltose, maltodextrin and pectin). The expression of certain secreted proteins is directly dependent on the sugar regulator TrmBL4, as is the ABC maltose/maltodextrin transporter (MD system), but not the trehalose/maltose transport system (TM system). Finally, the expression data for certain targeted genes show a possible synergy between TrmBL4 and TrmBL1.

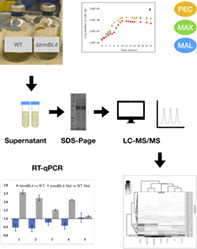

## INTRODUCTION

*Thermococcus barophilus* MP was the first true hyperthermophilic piezophile archaeon isolated from a deep‐sea hydrothermal vent (Marteinsson et al., 1999). Previous investigations have shown that its adaptation to high hydrostatic pressure (HHP) is achieved by the modulation of the expression of different gene clusters involved in energy, amino acid, and carbohydrate metabolism (Vannier et al., 2015). Notably, the maltose/maltodextrin ABC transporter (MD system) *TERMP*_(*01653–01658*) involved in carbohydrate metabolism is one of the HHP‐responsive gene clusters (Vannier et al., 2011). The expression of this MD system increases under high pressure, as does the expression of two other carbohydrate ABC transporters, *TERMP*_(*00275–00281*) and *TERMP*_(*01836–01840*; Vannier et al., 2015). Interestingly, the operon *TERMP*_(*00275–00281*) includes a transcriptional regulator gene (*TERMP*_*00277*) that encodes TrmBL4 (TrmB‐like protein 4), which is a family member of the global transcriptional regulator TrmB, known for its regulation of sugar metabolism. TrmB is especially known to be a repressor and/or activator of sugar ABC transporters and as a histone‐like chromosomal stabilizer in Archaea (Gindner et al., 2014; Kim et al., 2016).

Proteins of the TrmB family (TrmB, TrmBL1, TrmBL2, TrmBL3, TrmBL4, and TrmBL5) are present in 13 groups of Euryarchaeota (Lee et al., 2008). *Pyrococcus furiosus* has been shown to possess the genes *trmB* (*PF1743*), *trmBL1* (*PF0124*), *trmBL2* (*PF0496*), and *trmBL3* (*PF066*; Lee et al., 2008). Transcription assays show that TrmB of *P. furiosus* acts as a transcriptional repressor for the trehalose/maltose transport system (TM system) with high DNA binding affinity and for the MD system with lower affinity; this inhibition can be removed by the addition of specific sugars (Lee et al., 2005). Although, the TrmBL1 of *P. furiosus* acts as a repressor of genes of the MD system and glycolytic enzymes, and as an activator of genes involved in glucogenic pathways, it is inactivated by maltose and maltotriose (Lee, Surma, et al., 2007). Only preliminary experiments have been done on TrmBL2, which appears to recognize TM and MD promoters and to play a role at the DNA level, clumping it into dense fibrous structures (Maruyama et al., 2011). The TrmB family performs a multiplicity of regulations by acting on the same promoters: at least three regulators, TrmB, TrmBL1, and TrmBL2, regulate gene expression at the level of the MD promoter in *P. furiosus* (Lee et al., 2008). Interestingly, this species can grow on carbohydrates such as cellobiose, maltose, and starch (Kengen et al., 1993; Schmid et al., 2013), which implies the secretion of enzymes such as amylopullulanase (Schmid et al., 2013) and an α‐glycosidase (Constantino et al., 1990).

**FIGURE 1:**
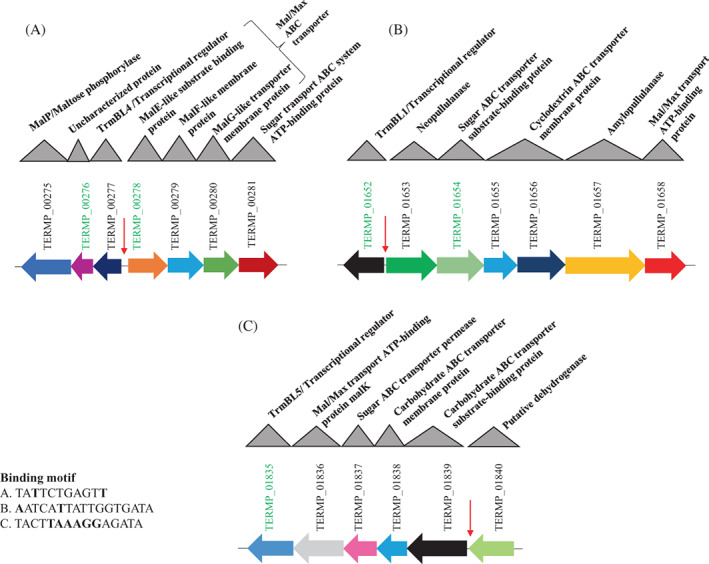
The organization of three clusters encoding genes involved in sugar metabolism and upregulated by high hydrostatic pressure in *Thermococcus barophilus*. (A) The *TERMP*_(*00275–00281*) cluster. (B) The *TERMP*_(*01652–01658*) cluster (MD system). (C) The *TERMP*_(*01835–01840*) cluster. The genes of interest representative of the target clusters for the reverse transcription quantitative polymerase chain reaction are highlighted in green. Red arrows indicate the TrmBL4 binding site and bold letters in the corresponding binding motifs indicate the non‐conserved bases. Mal/Max indicates maltose/maltodextrin.

The *trmB* gene in *Thermococcus litoralis* was characterized as a repressor of the TM system (Lee et al., 2003). The role of the other regulators (TrmBL3, TrmBL4, and TrmBL5) in the Thermococcales (Gindner et al., 2014) remains unclear. *Thermococcus barophilus* MP, the first true hyperthermophilic piezophile archaeon isolated from a deep‐sea hydrothermal vent (Marteinsson et al., 1999), possesses the *TERMP*_*01972* gene encoding TrmB, which might regulate the adjunct operon *TERMP*_(*01968–01973*; TM system); TrmBL2 encoded by the *TERMP*_*00762* gene; TrmBL4 encoded by the *TERMP*_*00277* gene, which can regulate the operon *TERMP*_(*00278–00281*) in the vicinity and divergently oriented (Figure 1A); TrmBL1 encoded by the *TERMP*_*01652* gene, which could regulate the operon *TERMP*_(*01653–01658*; MD system; Figure 1B); and TrmBL5 encoded by the *TERMP*_*01835* gene, which might be involved in the regulation of the *TERMP*_(*01835–01840*) cluster in *T. barophilus* (Figure 1C; Kim et al., 2016).

The binding sites of TrmB and TrmBL1 were identified in *P. furiosus* (TrmB: TACTN_3_AGTA for the TM system and TACT for the MD system; TrmBL1: TATCACN_5_GTGATA for the MD system) as was the binding site of TrmB in *T. litoralis* (TACTN_3_AGTA for the TM system; Lee et al., 2005). In 2006, Van de Werken et al. identified a conserved palindromic sequence, the Thermococcales glycolytic motif (TGM), TATCACN_5_GTGATA, present upstream of all genes involved in sugar metabolism in several Thermococcales species as well as in the promoter region of the MD operon, but not in the TM operon (Van de Werken et al., 2006). It has been shown that TrmB preferentially recognizes the TGM sequence (Lee, Seitz, et al., 2007), while TrmBL1 target binding is not restricted in this way because it also interacts with non‐TGM‐containing sequences with a lower binding affinity (Lee, Seitz, et al., 2007). TrmBL2 has no noticeable pattern with respect to other TGM‐containing promoters (Lee, Seitz, et al., 2007; Lee, Surma, et al., 2007). The binding site of TrmBL4, however, has not yet been studied.

Because little is known about the metabolism of sugar and its regulation in *T. barophilus*, we investigated the potential role of TrmBL4 through a reverse genetic approach in which its gene locus (*TERMP*_*00277*) was deleted. We then analysed the global secreted proteins, known as the ‘secretome’ of the wild‐type (WT) and mutant strains at 0.1 MPa in the presence of different carbohydrates. This made it possible, using a liquid chromatography with tandem mass spectrometry (LC–MS/MS) proteomic approach, to reveal different secretomes linked to the presence or absence of TrmBL4. Finally, we assessed the potential of TrmBL4 to regulate genes by performing reverse transcription quantitative polymerase chain reaction (RT‐qPCR) experiments on target genes involved in *T. barophilus* sugar metabolism.

## EXPERIMENTAL PROCEDURES

### Construction of mutants: Pop‐in/Pop‐out technique

The *TERMP*_*00277* locus (TrmBL4) was deleted by the Pop‐in/Pop‐out method to generate the *T. barophilus* Δ*trmBL4* strain following the protocol previously published in Birien et al. (2018) and Thiel et al. (2014) (Table 1).

**TABLE 1:** The list of specific primers used to delete the *trmBL4* gene using the Pop‐in/Pop‐out technique and to target genes of interest using the RT‐qPCR technique.

TrmBL4 primer	Primer sequence
KpnI‐D‐TrmBL4 1 Fwd	5′AAAAAAGGTACCGCTATCAGAGTTTTTAGAGTAGCATCTTCAATG 3′
D TrmBL4 1 Rev	5′ TGCAGTGAGGGGTGGTTAATAAAAGGAGGGATCTCCTTGAAAGCA 3′
D TrmBL4 2 Fwd	5′GGAGATCCCTCCTTTTATTAACCACCCCTCACTGCATTATATTCT 3′
BglII‐D TrmBL4 2 Rev	5′AAAAAAAGATCTATCTCCAAGCCATCCAGCACCGACA 3′

Target gene	Primer sequence
*TERMP*_*00276* Fwd	5′ATTGCCTCATTGTTAATGCCGTATTCTCC 3′
*TERMP*_*00276* Rev	5′ TTAATTATGTCTCCGGTAAGCCGCG 3′
*TERMP*_*00278* Fwd	5′ CCACCTACAACAGTGACAACAACAAA 3′
*TERMP*_*00278* Rev	5′ GGGGAATTACCTCATATTTAACGCCGA 3′
*TERMP*_*01652* Fwd	5′ GCTTAAGCAGGGTTATCAGCGTTCT 3′
*TERMP*_*01652* Rev	5′ TTGGAGCCCTTCCAAGGGATAGA 3′
*TERMP*_*01654* Fwd	5 GGAGGCGGAACAACCACTC 3′
*TERMP*_*01654* Rev	5′ GATTCAAAAACCTGCGCCTCATTTG 3′
*TERMP*_*01835* Fwd	5′ CCTTCAATCTCTACAGGCTCCTTGC 3′
*TERMP*_*01835* Rev	5′ TTGCTGTTCATGTTTGACAGGTACTTCT 3′
*TERMP*_*00342* (*pcna*) Fwd	5′ GCATGAGGGCAATGGATCCAA 3′
*TERMP*_*00342* (*pcna*) Rev	5′ GGGTTACCTCAAGGAAGTTCTCCTCA 3′
*TERMP*_*00128* (*30S*) Fwd	5′ GGCATAAACTTTGCCACGATGGTG 3′
*TERMP*_*00128* (*30S*) Rev	5′ TGCACCAATGAGGTGCATGTC 3′

*Note*: *TERMP*_*00276* and *TERMP*_*00278* representative of the *TERMP*_(*00275–00281*) cluster; *TERMP*_*01652* representative of the *TERMP*_(*01652–01658*) cluster; *TERMP*_*01654* representative of *TERMP*_ (*01652–01658*); *TERMP*_*01835* representative of *TERMP*_ (*01835–01840*); *TERMP*_*00342* and *TERMP*_*00128* used as control genes.

Abbreviation: RT‐qPCR, reverse transcription quantitative polymerase chain reaction.

### Culture medium and growth experiments with sugar

Cells of *T. barophilus* WT *Tb*Δ*517* (UBOCC‐M‐3300; Birien et al., 2018) and the derivative mutant *Tb*Δ*517*Δ*trmBL4* (UBOCC‐M‐3321) were cultivated under anaerobic conditions in Thermococcales rich medium (TRM; Zeng et al., 2009) at a 1× or 1.1× concentration (when sugars were added). The medium was supplemented with different sugar sources: maltose (maltose monohydrate, Sigma Lot no. SLCC3973), maltodextrin (maltodextrin, Sigma Lot no. MKCG8957), or pectin (pectin from citrus, Sigma Lot no. 97H0580) at a final concentration 0.5 g/L. The medium was then transferred into 50 mL penicillin vials with or without elemental sulphur (0.25 g/L), which acts as a terminal electron acceptor that enhances the growth of Thermococcales (Marteinsson et al., 1997). The cells were cultured at 85°C under P_atm_. To determine growth in each treatment, cells were counted using a Thoma counting chamber (0.01 mm depth, Weber, England) at *×*100 magnification (Olympus BX40).

### Recovery and concentration of extracellular proteins by ultrafiltration

To recover the secretome, the WT, and the mutant cells were cultured in 1‐L bottles with 330 mL TRM medium with and without sugars and then incubated at 85°C under P_atm_ with shaking at 200 rpm. As noted above, each of the three sources of sugar used was added to give a final concentration of 0.5 g/L except for the pectin, for which this was 0.15 g/L, because pectin interferes with the good separation and visualization of proteins. This minimum concentration of pectin was determined after several kinetic studies. For this, growth tracking of the cells was carried out in TRM medium in the presence of different concentrations of pectin (0.1, 0.15, 0.2, 0.25, 0.3, and 0.5 g/L) at 85°C (data not shown). Sulphur was added (0.25 g/L) to each treatment to enhance cell growth and thus obtain a sufficient cell concentration to study the secretome of the cells. The protocol for the concentration of extracellular proteins was modified from Schmid et al. (2013). The WT and mutant derivative cultures were collected at the late exponential phase (1–2 × 10^8^ cells/mL) and transferred to 450‐mL centrifuge beakers to be centrifuged for 5 min at 200 *× g*, 4°C, to remove the rest of the sulphur from the medium. The media were transferred to new beakers for further centrifugation for 1 h at 8000 *× g*, 4°C. The supernatants were then filtered on sterile filters (0.22‐μm pore size) to remove cell remains. The proteins in the supernatant fractions (350 mL) were concentrated by two ultrafiltration procedures; first, by using an Amicon Ultra 15 10 K ultrafiltration unit following the manufacturer's procedure (Merck Millipore‐Amicon® Ultra‐15 10 K Centrifugal Filter Devices) to obtain a final volume of 100 μL. A minimum molecular weight of 10 kDa was chosen after research carried out in silico showed that most of the proteins likely to be secreted by *T. barophilus* would be larger than 10 kDa. The second ultrafiltration procedure was performed using an Amicon ultra 2‐mL ultrafiltration unit. This filtration reduced the volume to 70 μL. To properly visualize proteins on an SDS‐PAGE gel, the concentration of the proteins present in the supernatant must be 1 mg/mL; therefore, the protein concentration in each treatment was determined by Bradford assay using a standard range of known concentrations (Schmid et al., 2013). Additionally, to correctly filter the potentially secreted proteins, the cytosolic proteins were also concentrated. For this, the recovered cell pellet was centrifuged at 8000 × *g* for 30 min at 4°C. Cell lysis was then performed by sonication by pulses of 0.5/0.5 s for 5 min at 4°C. Afterward, the sonicate was centrifuged at 10,000 × *g* for 30 min. The same protocol was applied to concentrate the cytosolic proteins used as a control.

### SDS‐PAGE and identification of proteins by MS

To visualize and separate proteins, the concentrated proteins from the supernatants of *T. barophilus* WT and mutant strains grown in the presence of sugars (maltose, maltodextrin, or pectin), were applied to a 4%–20% Tris‐Glycine Mini‐Protean gel (Bio‐Rad, Hercules, California). The deposited protein volume ranged from 10 to 20 μL, containing 1 mg/mL of proteins, although the final deposited volume needed to be the same for all tested conditions. To cater for this, phosphate‐buffered saline was used as an equilibration buffer and 4× XT sample buffer (Bio‐Rad no. 1610791) was used as a stain. The gel was stained with Coomassie Brilliant Blue R250 (0.2% wt/vol, 40% vol/vol isopropanol, and 7% vol/vol acetic acid). The gel was then destained with a mixture of 300 mL 97% ethanol and 100 mL glacial acetic acid in a total volume of 1 L. Gel lanes were sliced into pieces and sent to the PAPPSO platform (*Plateforme d'Analyse Protéomique de Paris Sud Ouest*) for LC–MS/MS analysis on a 1D gel track (shotgun); the cytosolic concentrated proteins were rather used as a control for the secretion process. The gel slices were digested with 200 ng trypsin, then the samples were dispersed in 20 μL loading buffer and 4 μL were injected on a Fusion Lumos Orbitrap Tribrid mass spectrometer. Quantitation of the proteins in all samples was performed using a Nanodrop 8000. Data processing was done with the X!TandemPipeline (0.4.40).

### Analysis of secreted proteins

To properly filter the potentially secreted proteins, two procedures were used. The first of these was the detection of signal peptides in silico with the OutCyte 1.0 program (Zhao et al., 2019). The second was the determination of the probability that a protein is secreted or not based on the results of MS analysis of the cytosolic proteins used as a control of secretion. In the calculations, detailed in Data S2, the determination of the secretion probability is based on the ratio of the amount of the protein in the supernatant to its presence in the cytosol while the cells are grown in the presence of the three tested sugars. Consequently, the secretion probability for each protein was determined and a heatmap was constructed based on a colorimetric scale showing the difference of normalized spectra (at 10,000/sample), which intuitively indicates the proteins that are the most abundant in the secretome of the tested samples. Hypothetical proteins with putative domain annotations were analysed using the Pfam 34.0 (Mistry et al., 2021) wrapper around the HMMER package, which analyses biosequences using hidden Markov models (Durbin et al., 1998), and the UCSC Archaeal Genome Browser database (Schneider et al., 2006). In the latter, the basic gene annotation is derived from the NCBI GenBank/RefSeq entries, with conservation overlays of sequences across multiple species and nucleotide/protein motifs, as well as non‐coding RNA and operon predictions.

The hierarchically clustered heatmap was constructed with the cluster map API of the statistical data visualization Python library *seaborn* (Waskom, 2021). The weighted linkage method was used for calculating clusters.

### Sample RNA extraction and complementary DNA synthesis

RNA was extracted with TRIzol. For this, the strains were pre‐cultured in a 20 mL TRM culture medium in the presence of sulphur, with and without maltose. WT and Δ*trmBL4* were inoculated at a rate of 2 × 10^6^ cells/mL of fresh *T. barophilus* pre‐culture, cultured for 6 h at 85°C at P_atm_, and harvested at the mid‐log phase by centrifugation at 5000 × *g* for 6 min at 4°C. The pellet was re‐suspended in 1 mL of Trizol and then transferred into 2 mL RNAse‐free tubes. Then, 200 μL chloroform was added, followed by shaking by hand. The mixture was left for 3 min at room temperature. 15‐min centrifugation was carried out at 12,000 × *g*, 4°C. Afterward, the aqueous phase was placed in a 1.5 mL RNAse‐free Eppendorf. To precipitate the RNA, 500 μL of isopropanol was added and the mixture incubated for 5 min at room temperature. The pellet was washed twice with 70% RNAse‐free ethanol followed by centrifugation for 15 min at 12,000 × *g*. After successive washings, the supernatant was removed, the residual ethanol dried out, and the RNA dissolved in water. Residual DNA was removed by DNase treatment (3 U of Turbo™ DNase, Invitrogen, according to the manufacturer's instructions). The quantity of extracted RNA was determined using a Nanodrop 8000 (Thermo Fisher Scientific Inc.) and it was stored at −80°C until use. The reverse transcriptions were performed using the iScript Reverse Transcription Supermix Kit for RT‐qPCR (Bio‐Rad) to obtain complementary DNA (cDNA) concentration of ~2.5 ng/μL. cDNAs were amplified (5 min at 25°C, 20 min at 46°C, and 1 min at 95°C).

### Reverse transcription quantitative polymerase chain reaction

Quantitative PCR analysis was carried out on three clusters of genes, each with a representative gene, and primers were designed using SnapGene software (Figure 1 and Table 1). qPCR reactions with *pcna* and the *30S* gene were used as an internal control. Three biological replicates were performed for each treatment. Data were recorded and processed using Bio‐Rad CFX Maestro software. The analysis was performed using the ΔΔCt method to calculate expression changes compared with the control (Livak & Schmittgen, 2001; Schmittgen & Zakrajsek, 2000). The control gene corresponds to a gene in the genome expressed under all growth conditions and is, therefore, unregulated. This means that the expression of this gene should be relatively stable under all the studied conditions.

## RESULTS

### Thermococcus barophilus *growth enhancement by carbohydrates*

**FIGURE 2:**
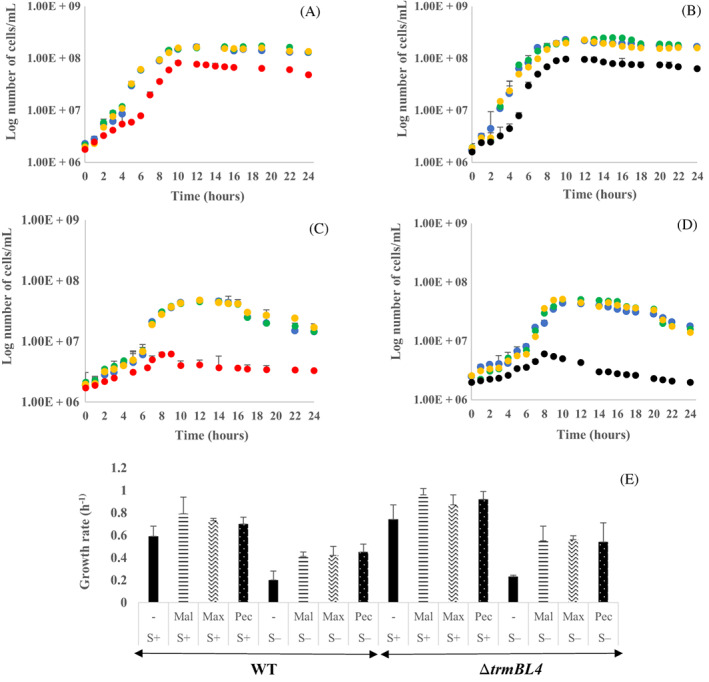
Tracking the growth of the wild‐type (WT) and the Δ*trmBL4* cells with or without sugars in the presence and absence of sulphur at 0.1 MPa. WT cells cultured without sugars (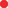); Δ*trmBL4* cells cultured without sugars (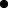); cells cultured in the presence of maltose (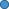), maltodextrin (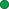), or pectin (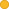). (A) The growth of the WT cells in the presence and absence of sugars in the presence of sulphur. (B) The growth of Δ*trmBL4* cells in the presence and absence of sugars in the presence of sulphur. (C) The growth of the WT cells in the presence and absence of sugars in the absence of sulphur. (D) The growth of Δ*trmBL4* cells in the presence and absence of sugars in the absence of sulphur. (E) Comparison of the growth rate of the WT and Δ*trmBL4* cells in the presence of maltose (Mal), maltodextrin (Max), and pectin (Pec), in the presence and absence of sulphur at 0.1 MPa. A different pattern is used for each type of sugar for both WT and Δ*trmBL4* cells. A solid fill is used where no sugar was added. The growth rate, which is the inverse of generation time (G), represents the number of generations per unit of time in an exponentially growing culture. It is equal to 1/G, expressed in inverse units of time (h^−1^). The generation time (G) represents the time necessary for the doubling of a population in the exponential phase. Error bars represent standard deviations obtained from at least three biological replicates with three technical replicates each.

To test the ability of *T. barophilus* to metabolize carbohydrates, we assessed its growth kinetics in TRM medium with or without the sugars maltose (MAL), maltodextrin (MAX), or pectin (PEC), each at a final concentration of 0.5 g/L. We compared the growth of the derivative mutant Δ*trmBL4* strain against the WT to explore the potential role of the uncharacterized TrmB‐like family regulator. Knowing the critical role of sulphur in Thermococcales growth (Bertoldo & Antranikian, 2006), the cultures were also grown in the presence (Figure 2AB) or absence | (Figure 2C,D) of elemental sulphur (0.25 g/L).

Our results show that growth is slightly enhanced in the presence of any sugar source, with an increase in the number of the WT cells, reaching a cell yield of 1.6 × 10^8^ cells/mL at stationary phase (Figure 2A, blue dots, with sulphur), and a growth rate of 0.79 h^−1^ with maltose (Figure 2E). This compares with 8 × 10^7^ cells/mL (Figure 2A, red dots, with sulphur) and a growth rate of 0.59 h^−1^ in the absence of sugars. In the case of the Δ*trmBL4* cells, the cell yield reached 2.5 × 10^8^ cells/mL at stationary phase (Figure 2B, blue dots, with sulphur), with a growth rate of 0.96 h^−1^ with maltose (Figure 2E), compared with 9.1 × 10^7^ cells/mL (Figure 2B, black dots, with sulphur) and a growth rate of 0.74 h^−1^ without any sugar (Figure 2E). The difference in cell concentration between the treatments with and without sugars was more pronounced when sulphur was absent from the medium. Cell growth in the presence of sugars reached 4.3 × 10^7^ and 7 × 10^7^ cells/mL, with growth rates of 0.42 and 0.56 h^−1^, in WT and Δ*trmBL4* cells, respectively, in the presence of maltodextrin (Figure 2C,D, green dots, and Figure 2E without sulphur) compared with ~6 × 10^6^ cells/mL for both strains in the absence of sugars (0.2 h^−1^ growth rate; Figure 2C,D, red/black dots and Figure 2E without sulphur). Furthermore, a boost in the growth of the mutant cells compared with the WT strain was detected in the presence of maltose and sulphur (blue dots on Figure 2A,B, respectively), as the cell yields reached 2.5 × 10^8^ cells/mL in Δ*trmBL4* at stationary phase but only 1.6 × 10^8^ cells/mL in WT. These improvements in cell growth suggest the secretion of enzymes capable of degrading carbohydrates in the culture medium.

### Extracellular proteins detected in the supernatant

**FIGURE 3:**
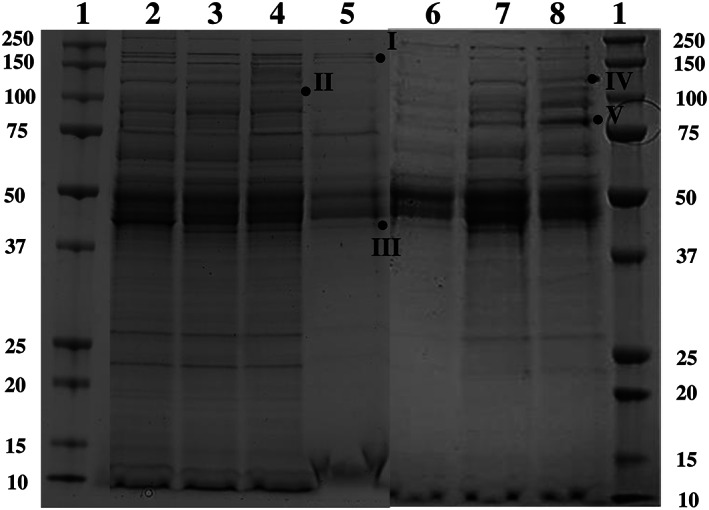
A representative Coomassie blue stained SDS‐PAGE gel showing concentrated proteins from *Thermococcus barophilus* wild‐type (WT) and Δ*trmBL4* cell culture supernatants. 1. PageRuler™ Prestained Protein Ladder; molecular weights are indicated in kDa; 1 mg/mL of proteins from each culture supernatant was loaded. 2. WT culture in the absence of sugars. 3. WT culture in the presence of maltose. 4. WT culture in the presence of maltodextrin. 5. WT culture in the presence of pectin. 6. Δ*trmBL4* culture in the absence of sugars. 7. Δ*trmBL4* culture in the presence of maltose. 8. Δ*trmBL4* culture in the presence of maltodextrin. A few additional bands detected in the presence of sugars are indicated by Roman numerals.

**FIGURE 4:**
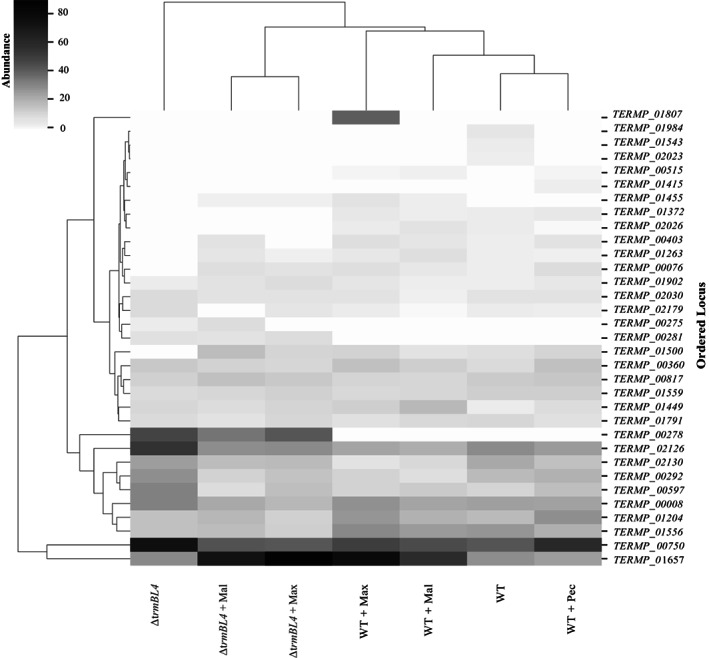
Hierarchical heat map for the visualization of the differential expression of the secreted proteins in each sample. The heat map was produced with the *seaborn* program using the liquid chromatography with tandem mass spectrometry data and shows only the secreted proteins (Data S3) from Data S1. The samples are shown in the columns and the ordered loci for the genes encoding the secreted proteins in the rows. Columns are centred, with relative abundance (the difference of normalized spectra) represented by the darkness of shading (white: lower abundance; black: higher abundance) of the secreted proteins in the tested conditions. Mal, Max, and Pec indicate maltose, maltodextrin, and pectin, respectively.

Cells from the WT and Δ*trmBL4* strains were grown in TRM medium supplemented with carbohydrates (maltose 0.5 g/L, maltodextrin 0.5 g/L, or pectin 0.15 g/L), in the presence of sulphur (0.25 g/L), to obtain sufficient proteins for testing and analysis. Cells were harvested at the end of the exponential growth phase (10 h) and their supernatant fractions were collected. The concentrated proteins were separated by 1D‐gel electrophoresis (Figure 3), cut into slices, and sent to the PAPPSO platform for MS analysis, as were the cytosolic proteins used as a control. There, the proteins were analysed using high‐resolution LC–MS/MS. A few additional proteins were detected on the gel in the case where the WT and mutant cells were cultured in the presence of sugars (indicated on the gel, Figure 3). For example, a protein of 44 kDa molecular weight was present in the secretome of the WT cells cultured in the presence of any sugar but absent when no sugars were provided (Figure 3, Band III). Another protein, 120 kDa, was only detected in the secretome of the WT cells in the presence of maltodextrin (Figure 3, Band II). In addition, a protein of 87 kDa was detected in the secretome of the mutant cells in the presence of sugars but was absent when none were provided (Figure 3, Band V). Despite the decrease in the concentration of pectin (0.15 g/L instead of 0.5 g/L) for the proteomic analysis, as explained in the experimental procedures section, technical issues were encountered when preparing proteins from the supernatant of the WT cells grown in the presence of pectin (data not shown). Consequently, the secretome of Δ*trmBL4* cells in the presence of pectin was not studied. To obtain a description of the dataset generated by the LC–MS/MS, a hierarchical clustering heatmap was constructed based on the values, showing the difference of normalized spectra (at 10,000/sample). This method reveals which of the secreted proteins are the most abundant in the secretome of the tested samples (Figure 4 and Data S3). The detected cytosolic proteins were removed from the overall data (Data S1) to focus on the secretome. A total of at least 33 proteins were secreted in the supernatant of the WT and mutant cells cultured in the presence of sugars (Figure 4, Table 2, and Data S2). The corresponding heatmap highlights a WT‐mutant dichotomy. Figure 4 shows the mutant cells on the left and the WT cells on the right, cultivated in the presence or absence of sugars. With the added carbohydrates, we see a clear shift in the secreted proteins of the mutant cells, with only some slight differences between maltose and maltodextrin. This did not happen with the WT cells, where notable differences could be observed, for example, the protein encoded by *TERMP*_*01807* (S‐layer domain) was only expressed in the WT cells with maltodextrin. Interestingly, no common effect was detected between the three sugars, which could be because pectin may induce another secretome closer to the one that appears without sugar than to those with maltose or maltodextrin.

**TABLE 2:** The list of secreted proteins identified in the supernatant of *Thermococcus barophilus* wild‐type and Δ*trmBL4* cells grown on sugars.

Ordered locus	Found only in the supernatant	Secretion probability	N‐terminal signal sequence^a^	Molecular weight	Description	Pfam/UCSC archaeal genome^b^	TrmBL4 binding motif^c^
*TERMP*_*01807*	Yes	0.5	Yes	101.5	S‐layer domain		No
*TERMP*_*01984*	Yes	1	Yes	68.1	Lipoprotein		No
*TERMP*_*01543*	Yes	1	Yes	45.2	Uncharacterized protein	Eight‐cysteine‐cluster domain	No
*TERMP*_*02023*	Yes	1	Yes	22	Flagellin		Yes
*TERMP*_*00515*	Yes	0.5	Yes	70.5	S‐layer_C domain‐containing protein		No
*TERMP*_*01415*	Yes	0.5	Yes	71.6	Uncharacterized protein	Hypothetical protein	No
*TERMP*_*01455*	Yes	0.5	Yes	81	Cyclomaltodextrin glucanotransferase		No
*TERMP*_*01372*	Yes	1	Yes	56.4	Uncharacterized protein	Amylopullulanase	No
*TERMP*_*02026*	Yes	1	Yes	51	Uncharacterized protein	Hypothetical protein	No
*TERMP*_*00403*	Yes	1	No	8.9	RNA‐binding protein		No
*TERMP*_*01263*	Yes	1	No	27.7	Metal‐dependent hydrolase		No
*TERMP*_*00076*	Yes	1	No	18.8	Phosphoesterase		No
*TERMP*_*01902*	Yes	1	No	17.6	Peptidyl‐prolyl cis‐trans isomerase		No
*TERMP*_*02030*	Yes	1	No	22	GMP synthase		No
*TERMP*_*02179*	Yes	1	Yes	209.5	Peptidase_S8 domain‐containing protein		No
*TERMP*_*00275*	Yes	0.5	No	86.8	Maltose phosphorylase		No
*TERMP*_*00281*	Yes	0.5	No	40.1	Sugar ABC transporter		No
*TERMP*_*01500*	Yes	1	No	8.2	RNA‐binding protein		Yes
*TERMP*_*00360*	No	1	No	21.2	DNA protection during starvation protein		No
*TERMP*_*00817*	No	1	No	21.5	Thymidine kinase		No
*TERMP*_*01559*	No	1	No	12	Ketoisovalerate oxidoreductase subunit		No
*TERMP*_*01449*	Yes	1	Yes	76.4	Dockerin domain‐containing protein		No
*TERMP*_*01791*	No	1	Yes	48.5	S‐layer‐like array protein		No
*TERMP*_*00278*	Yes	0.5	Yes	50.9	MalE‐like Mal/Max ABC transporter		No
*TERMP*_*02126*	No	1	Yes	179.8	vWFA domain‐containing protein		No
*TERMP*_*02130*	Yes	1	Yes	21.8	Methyltransferase		No
*TERMP*_*00292*	No	1	Yes	54.3	PEGA domain‐containing protein		Yes
*TERMP*_*00597*	No	1	Yes	80.6	NPCBM_assoc domain‐containing protein	Domain of α‐ galactosidase	No
*TERMP*_*00008*	No	1	No	38.5	Glycosidase		No
*TERMP*_*01204*	No	1	Yes	22.8	Flagellin		No
*TERMP*_*01556*	No	1	No	12	Pyruvate: ferredoxin oxidoreductase Δsubunit		No
*TERMP*_*00750*	No	1	Yes	76.3	CARDB domain‐containing protein		No
*TERMP*_*01657*	No	1	Yes	153.6	Amylopullulanase		Yes

*Note*: The supernatant only represents the proteins found only in the supernatant, not in the cytosol; the secretion probability is calculated according to the equations in Data S2 and Mal/Max for maltose/maltodextrin.

^a^ Prediction of N‐terminal signal sequence using the OutCyte 1.0 program (Zhao et al., 2019).

^b^ Significant hit obtained after a search in Pfam 34.0 (Mistry et al., 2021) and the UCSC Archaeal Genome Browser database for hypothetical proteins (Schneider et al., 2006).

^c^ Search for the TrmB‐like binding motif performed in silico on the whole genome of *T. barophilus*, based on the known TrmB binding motifs in certain Thermococcales.

Among the proteins of the secretome, several were repressed in the mutant cells under particular conditions. These included a protein encoded by *TERMP*_*01556*, a pyruvate ferredoxin oxidoreductase Δ‐subunit catalysing one of the final steps in carbohydrate fermentation (Blamey & Adams, 1993), which was found to be repressed in the mutant cells regardless of sugar availability. Two hypothetical proteins, respectively encoded by *TERMP*_*01372* and *TERMP*_*02026*, were also repressed in the mutant. Conversely, we detected an overexpression of some proteins in the mutant strain. These included the protein encoded by *TERMP*_*02130*, which was expressed slightly more in the mutant cells than in the WT cells; and the protein encoded by *TERMP*_*00597*, hypothetically coding for an alpha‐galactosidase NEW3 domain, whose expression was higher in the mutant cells cultured without sugars. Additionally, the proteins encoded by *TERMP*_*00275*, *TERMP*_*00281*, and *TERMP*_*00278*, which are part of the HHP‐responsive cluster and close to the TrmBL4 regulator (Vannier et al., 2015), were also overexpressed and only detected in the case of the mutant. However, the protein encoded by *TERMP*_*01657*, coding for an amylopullulanase that is part of the second HHP‐responsive cluster *TERMP*_(*01653–01658*; MD system), was overexpressed in both the WT and mutant secretomes in the presence of maltose and maltodextrin, whereas the other proteins of this operon were only slightly overexpressed in the mutant in the presence of sugars (Data S1). Regarding the *TERMP*_(*01836–01838*) operon, no expression was detected.

Surprisingly, the proteins encoded by *TERMP*_*00360* and *TERMP*_*02126* were expressed in all tested conditions. The first of these, corresponding to DNA protection during starvation protein, is known to protect DNA during nutrient starvation and against oxidative DNA damage (Frenkiel‐Krispin et al., 2001). The second corresponds to a vWFA domain‐containing protein known to be involved in cell adhesion, signal transduction, extracellular matrix proteins, and integrin receptors (Whittaker & Hynes, 2002).

Furthermore, the expression of the hypothetical proteins encoded by *TERMP*_*01984*, *TERMP*_*01543*, and *TERMP*_*02023* was altered by both the mutation and the presence of sugars because they are expressed only in the WT cells in the absence of sugars. In contrast, we detected the presence of a cyclomaltodextrin glucanotransferase, encoded by *TERMP*_*01455*, whose expression is controlled by the presence of maltose and maltodextrin in the medium independently of the mutation.

In addition, we observed that the expression of putative RNA binding proteins was altered the proteins encoded by *TERMP*_*00403* and *TERMP*_*01500* were repressed in the mutant cells compared with the WT cells in the absence of sugars. This was also the case with the phosphoesterase encoded by *TERMP*_*00076*, whose expression was activated in the mutant cells by the addition of sugars. These proteins were found in the supernatant of the cells but had no detectable signal peptide.

### Relative expression of genes in the presence and absence of sugars studied by RT‐qPCR

**FIGURE 5:**
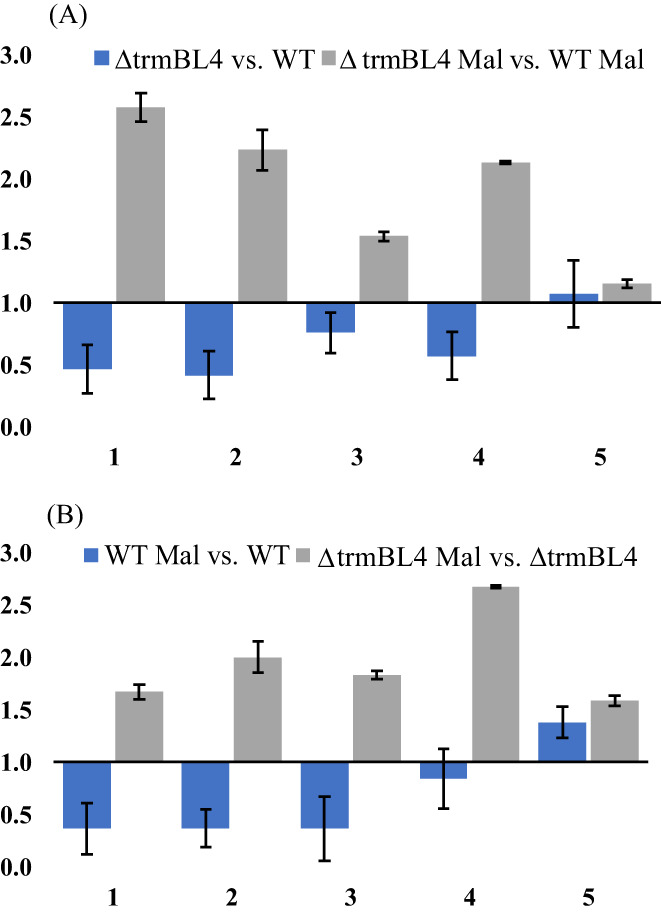
Comparison of the relative expression of the genes of interest representative of the target clusters under different conditions in the presence and absence of maltose, showing the effect of *trmBL4* gene mutation (A) and the effect of sugar (B). 1. and 2. *TERMP*_*00276* and *TERMP*_*00278* representative of the *TERMP*_(*00275–00281*) cluster; 3. *TERMP*_*01652* representative of the *TERMP*_(*01652–01658*) cluster; 4. *TERMP*_*01654* representative of *TERMP*_ (*01652–01658*); 5. *TERMP*_*01835* representative of *TERMP*_(*01835–01840*).

To determine the potential consequences of the deletion of the *trmBL4* gene for the expression of the three HHP‐responsive gene clusters involved in the metabolism of sugars in *T. barophilus*, we performed RT‐qPCR experiments targeting some representative genes of clusters of interest determined by the results of the LC–MS/MS analysis and the transcriptomics data published by Vannier et al. (2015). These genes were *TERMP*_*00276* and *TERMP*_*00278*, representative of the *TERMP*_(*00278–00282*) cluster (Figure 1A); *TERMP*_*01652* and *TERMP*_*01654*, representative of the *TERMP*_(*01652–01658*) cluster (Figure 1B); and *TERMP*_*01835*, representative of the *TERMP*_ (*01835–01840*) cluster (Figure 1C). The effects of the deletion of the *trmBL4* gene and of sugar addition were tested (Figure 5A,B, respectively). When comparing the results of Δ*trmBL4* and WT cells cultivated in the absence of maltose (Figure 5A), a repression of the expression of the representative genes of *TERMP*_(*00275–00281*) and *TERMP*_(*01652–01658*) was observed in the mutant strain, implying that *trmBL4* acts as an activator of the expression of the corresponding genes in the absence of maltose. Moreover, the results of WT cells cultivated in the presence or absence of maltose highlight repression of the same representative genes in the presence of maltose (*TERMP*_*00276*, *TERMP*_*00278*, *TERMP*_*01652*, and *TERMP*_*01654*). Comparison between the results of Δ*trmBL4* and WT showed that, in the presence of maltose, the expression of the representative genes of *TERMP*_(*00275–00281*) and *TERMP*_(*01652–01658*) was increased in the mutant strain, linking TrmBL4 as a repressor of the gene expression. So far, the operon *TERMP*_(*00275–00282*) seemed to be regulated by TrmBL4. The deletion of the *trmBL4* gene once again allowed, in the presence of maltose, an increase in the relative expression of this operon (Figure 5B). Concerning the expression of *TERMP*_*01654*, the representative gene of the *TERMP*_(*01652–01658*) operon, the increase was smaller, probably because of a lower affinity of the *trmBL4* regulator on this operon. In contrast, the expression of *TERMP*_*01835*, the representative gene of the *TERMP*_(*01835–01840*) cluster, was not affected by TrmBL4 and was only slightly impacted by the addition of sugar.

### Identification of TrmBL4 DNA binding domains

To check for a connection between TrmBL4 and the genes that seem to be controlled by this regulator, an in silico screening for the TrmB‐like binding motif was performed on the whole genome of *T. barophilus* based on the known binding motifs of TrmB and TrmBL1 discovered in other Thermococcales (Lee et al., 2008; Lee, Surma, et al., 2007). We detected a high degree of conservation between the binding motifs in *T. barophilus* compared with those known in *P. furiosus*. Concerning the *Pfu*‐TrmB binding site, the same TACTN_3_AGTA sequence was found in *T. barophilus* for the TM system, except for two nucleotides that differ from the *Pfu*‐TrmBL1 binding site for the MD system (TATCACN_5_GTGATA in *P. furiosus* and **A**ATCA**T**N_5_GTGATA in *T. barophilus*).

A binding site (**A**ATCA**T**N_5_GTGATA) was found upstream of *TERMP*_*01653* (MD system) at position 1,439,024–1,439,040. This binding motif includes the second half of the TGM sequence **GTGATA**. Moreover, the binding site (TACTN_3_AGTA) was detected upstream of *TERMP*_*01968* (TM system) at the positions 1,762,048–1,762,068 and 1,762,230–1,762,240. Two additional binding sites (TA**T**TCTGAGT**T,** pos. 232,136–232,146 and TACT**N**_**6**_AG**A**TA, pos. 1,624,822–1,624,836) were detected upstream of *TERMP*_*00278* and *TERMP*_*01840*. Three additional binding sites (TA**C**T**T**AGTT, pos. 1,295,540–1,295,548; TA**C**T**G**CTT, pos. 1,806,176–1,806,183; and TACT**CN**_**3**_ **CA**TA, pos. 246,815–246,826) were detected upstream of *TERMP*_*01500*, *TERMP*_*02023*, and *TERMP*_*00292*, respectively. In addition, a complete TGM motif was detected upstream of *TERMP*_*01654* (MD system) at position 1,441,219–1,441,235. Surprisingly, no TGM sequence was detected upstream of the *TERMP*_(*00278–00281*) operon or in other regulated glycolytic genes.

## DISCUSSION

Little is known about the secretome of *T. barophilus*, which plays an important role in sugar uptake and metabolism. In this study, we made the first analysis of the carbohydrate metabolism in *T. barophilus* by studying its secretome when cultured in the presence of carbohydrates at P_atm_ using genetic, physiological, and proteomics approaches. Our work represents the first step towards deciphering the organism's mechanism of adaptation to HHP. The regulation of sugar ABC transporters was assessed using deletion mutants of the transcriptional regulator of the TrmB family (TrmBL4) present in *T. barophilus*, which is known to affect the transcription of genes involved in sugar metabolism in Thermococcales. The effect of the deletion of the *trmBL4* gene was reflected by the growth enhancement of the mutant cells compared with the WT in the presence of sugars and these improvements were more pronounced in the absence of sulphur. Regardless of the mutation, the cells in a sulphur‐free medium relied more on the sugars to grow and seemed to use sugars as a source of carbon for growth. These results are supported by studies showing that sulphur may play a role in the metabolism of sugars in Archaea (Adams et al., 2001).

The proteomic results highlight critical proteins of the *T. barophilus* secretome, which are probably at least regulated by the *trmBL4* gene product, and others might be impacted by cultivation under non‐optimal growth pressure in *T. barophilus*. The STRING database search for protein–protein interactions (Szklarczyk et al., 2019) of the hypothetical amylopullulanase encoded by *TERMP*_*01372*, repressed in the mutant cells, revealed that the adjacent interacting proteins correspond to a secretion system. This probably indicates a new sugar‐related secretion system under the control of the *trmBL4* gene product. Additionally, the interacting proteins encoded by *TERMP*_*02026* and *TERMP*_*02023*, repressed in the mutant cells, could have formed a cross‐network regulating the cell shape and controlling the timing of the cell division in our tested conditions under the control of the *trmBL4* gene. This is likely because the adjacent interacting proteins, such as a tubulin cell division FtsZ‐like protein (*TERMP*_*02021*), are involved in the control of cell division.

Surprisingly, the protein responsible for pyruvate conversion was expressed under all conditions, with just slight repression in the mutant cells. The presence of this enzyme can be explained either by the use of pyruvate to cope with non‐optimal pressure or the activation of the conversion of pyruvate due to its accumulation in the cell. This pyruvate could thus be mobilized under stressful pressure conditions, as in the case of glutamate biosynthesis in *Thermococcus piezophilus* (Moalic et al., 2021).

Additionally, some proteins were found to be overexpressed in the mutant cells. These included a methyltransferase encoded by *TERMP*_*02130*, which may play a role in controlling the profile of gene expression impacted by the mutation. In addition, the search made in the STRING database (Szklarczyk et al., 2019, 2023) on another overexpressed key protein encoded by *TERMP*_*00597*, showed that the adjacent interacting proteins correspond to ABC transporters, which may indicate their disposition in a potential operon involved in sugar transport.

Contrastingly, the expression of the DNA protection during starvation protein and the vWFA domain‐containing protein in all tested conditions may indicate a stress response due to the alteration of nutrient uptake under non‐optimal pressure conditions (0.1 MPa) and modulation of the cell signalling pathways at 0.1 MPa, respectively.

Both the *trmBL4* gene mutation and the carbohydrates were able, nonetheless, to repress the expression of the protein encoded by *TERMP*_*01543* as though, in the absence of sugars, the presence of the *trmBL4* gene activates its expression. Therefore, we took a closer look at this protein to decipher its role. *TERMP*_*01543* may contain an eight‐cysteine‐cluster domain that could play a role in the biofilm formation as in the fungus *Candida albicans* (Pérez et al. 2006). Indeed, it is known that Archaea biofilms are formed by extracellular polymeric substances composed of polysaccharides, proteins, and metal ions (Stoodley et al., 2002). Studies have indicated that biofilm formation in Archaea provides benefits to the cells, including protection against stressful growth conditions such as toxic chemicals and low or high pH (Koerdt et al., 2010; Megaw & Gilmore, 2017). The phenotypic shifts in the behaviour of Archaea caused by stressful growth conditions require many regulatory processes and lead to the development of biofilms (An & Parsek, 2007). Here, in *T. barophilus*, the mutation of the *trmBL4* gene, addition of sugars, and growth under non‐optimal pressure conditions may have altered the biofilm formation by repressing genes exhibiting an enhanced ability to form biofilms. In *S. solfataricus*, the products of β‐galactosidase and α‐mannosidase can control the saccharide volume in the biofilm matrix (Koerdt et al., 2010).

Based on the proteomic results and the KEGG pathway database (Kanehisa, 2002), it is clear that maltose and maltodextrin share some enzymes and thus some common pathways, but pectin may not share the main assimilation systems with maltose and maltodextrin due to its complexity. Pectin is known to be degraded by specific pectinolytic enzymes with the corresponding transporters, as in the pectin degradation pathway in *Erwinia chrysanthemi* (now reclassified as *Dickeya dadantii*; Hugouvieux‐Cotte‐Pattat et al., 1996).

The S‐layer domain protein encoded by *TERMP*_*01807*, which was only overexpressed in the presence of maltodextrin in the WT cells, is probably involved in a specific maltodextrin membrane transport system because its adjacent interacted protein encoded by *TERMP*_*01808* corresponds to a major facilitator superfamily transporter. Additionally, we think that the expression of proteins forming part of the S‐layer domain is also pressure‐dependent, highlighting their role in cell stabilization under sub‐optimal pressure (0.1 MPa) since *TERMP*_*01807* was over‐expressed at 0.1 versus 40 MPa (Vannier et al., 2015). This phenomenon was also observed in the gene expression patterns of *T. piezophilus* (Moalic et al., 2021).

Intriguingly, the expression of RNA‐binding proteins requires the addition of sugars in the mutant cells, but not the WT cells. In other words, the presence of the *trmBL4* gene compensates for the absence of sugar. These proteins seem to have a role in regulating gene expression in our different tested conditions (Murina & Nikulin, 2011).

The presence of proteins in the supernatant of the cells devoid of a signal peptide can be explained by cell lysis, the limit of detection of the OutCyte program, or by them being classified as moonlighting proteins (Jeffery, 2003; Huberts & Van der Klei, 2010), which are uni‐functional proteins able to operate on both intracellular and extracellular levels (Sengupta et al., 2008). Particularly in the case of Archaea, another possible explanation is the export of proteins via membrane vesicles (Ellen et al., 2009; Ellen et al., 2010; Soler et al., 2008). More work is needed to determine which of these possible explanations are valid.

Interestingly, the RT‐qPCR results highlight a particular regulatory mechanism of the *trmBL4* gene product on some HHP‐responsive gene clusters involved in carbohydrate metabolism. In the absence of maltose, TrmBL4 seems to activate gene expression but, in the presence of this sugar, it acts as a repressor of gene expression. In our case, sugars did not rescue transcription as seen in *Pfu*‐TrmBL1 (Lee et al., 2008; Lee, Surma, et al., 2007). So, in the presence of maltose, the *trmBL4* regulator seems to act as a global transcriptional repressor of the *TERMP*_(*00275–00281*) cluster, with high binding affinity, and of the *TERMP*_(*01652–01658*) cluster (MD system), with lower affinity, defining it as a regulon. It should also be mentioned that the regulation of the expression of genes is not directly related to the composition of the secretome. The absence of TrmBL4 can have many more repercussions than those observed on the target genes tested by RT‐qPCR, such as effects on other target genes, retro‐control, and protein–protein interactions, which could all consecutively modify the secretome of *T. barophilus*.

In particular, the LC–MS/MS and RT‐qPCR results may indicate synergy between TrmBL4 and TrmBL1, since each of these may regulate the MD operon but with different affinities. Indeed, the expression of the *trmBL1* gene encoded by *TERMP*_*01652* is increased in the mutant, as shown by the LC/MS–MS (Data S1) and the RT‐qPCR results, as though compensating for the mutation of one of the regulators, the *trmBL4* gene. Consequently, we propose that these two members of the TrmB family are involved in a cross‐regulatory network of sugar‐sensing transcriptional regulators, as in the case of *P. furiosus*, characterized by the multiplicity of regulators acting on the MD promoter (Lee et al., 2008). Thus, in the absence of maltose, the TrmBL4 regulator alone may repress its expression leading to the de‐repression of the targeted genes, but repression is otherwise activated by maltose. This would mean that maltose stimulates the synthesis of TrmBL4, which would in turn repress the expression of genes encoding glycolytic enzymes. This emphasizes the important role of sugars in controlling transport and glycolysis. Moreover, TrmBL4 had no detectable effect on the *TERMP*_(*01835–01840*) operon. Interestingly, this operon was previously found to be overexpressed at high pressure (40 MPa; Vannier et al., 2015) but was not detected at 0.1 MPa.

The *in*‐*silico* screening for TrmBL4 binding sites indicates a certain degree of conservation, showing a divergence linked to the specific evolution of TrmBL4 compared with other TrmB‐like regulators. The latter need to be studied by footprint analysis on the promoter regions. The TrmBL4 target binding site also does not seem to be restricted to a TGM sequence because it is also involved in the regulation of genes with non‐TGM‐containing sequences.

## CONCLUSION

This study presents the first description of sugar metabolism gene products in *T. barophilus* and the potential regulation of some of these by the *trmBL4* gene product at atmospheric pressure. Many valuable proteins have been identified that could be the subject of further molecular studies in the near future. Some of the gene clusters coding for these proteins appear to be under the control of TrmBL4, one of the five TrmB‐like transcriptional regulators in the *T. barophilus* genome. It would be interesting to compare the composition of the secretome in the presence of sugars according to the presence or not of sulphur. It seems that, in a free sulphur medium, the sugars participate more actively in the energy metabolism and should therefore be more assimilated by the cells. Also, the regulation of the expression of the genes by TrmBL4 described in this study needs to be carried out at HHP because some genes of sugar metabolism are known to be responsive to these conditions. This is the first study of a TrmB family regulator in *T. barophilus*, opening up the possibility of a subsequent in‐depth study to characterize the gene expression regulation mechanisms of sugar metabolism structurally, molecularly, genetically, and biochemically in relation to hydrostatic pressure.

## AUTHOR CONTRIBUTIONS

**Maria Batour:** Methodology (equal); writing—original draft (equal). **Sebastien Laurent:** Data curation (equal); methodology (equal); writing—review and editing (equal). **Yann Moalic:** Formal analysis (equal); methodology (equal); writing–review and editing (equal). **Hala Chamieh:** Supervision (supporting); writing—review and editing (equal). **Samir Taha:** Writing—review and editing (supporting). **Mohamed Jebbar:** Conceptualization (lead); funding acquisition (lead); project administration (lead); resources (lead); supervision (lead); validation (lead); writing—original draft (lead); writing—review and editing (lead).

## CONFLICT OF INTEREST STATEMENT

The authors declare no conflicts of interest.

## Supporting information


**Data S1:** list of *T. barophilus* proteins (secreted and cytosolic) identified by LC/MS–MS.

**Data S2:** the list of proteins most likely secreted by *T. barophilus* resulting from semi‐quantitative LC/MS–MS analyses.

**Data S3:** LC/MS–MS analysis of secreted proteins in *T. barophilus* and the effect of carbohydrates (Maltose, Maltodextrins, and pectin) on their expression level.

## Data Availability

Datasets can be found in the Supplementary Data files.

## References

[emi413186-bib-0001] Adams, M.W. , Holden, J.F. , Menon, A.L. , Schut, G.J. , Grunden, A.M. , Hou, C. et al. (2001) Key role for sulfur in peptide metabolism and in regulation of three hydrogenases in the hyperthermophilic archaeon *Pyrococcus furiosus* . Journal of Bacteriology, 183(2), 716–724. 11133967 10.1128/JB.183.2.716-724.2001 PMC94929

[emi413186-bib-0002] An, D. & Parsek, M.R. (2007) The promise and peril of transcriptional profiling in biofilm communities. Current Opinion in Microbiology, 10, 292–296. 17573234 10.1016/j.mib.2007.05.011

[emi413186-bib-0003] Bertoldo, C. & Antranikian, G. (2006) The order Thermococcales. In: Dworkin, M. , Falkow, S. , Rosenberg, E. , Schleifer, K.H. & Stackebrandt, E. (Eds.) The prokaryotes. New York, NY: Springer.

[emi413186-bib-0004] Birien, T. , Thiel, A. , Henneke, G. , Flament, D. , Moalic, Y. & Jebbar, M. (2018) Development of an effective 6‐methylpurine counterselection marker for genetic manipulation in *Thermococcus barophilus* . Genes, 9(2), 77. 29414865 10.3390/genes9020077 PMC5852573

[emi413186-bib-0005] Blamey, J.M. & Adams, M.W. (1993) Purification and characterization of pyruvate ferredoxin oxidoreductase from the hyperthermophilic archaeon *Pyrococcus furiosus* . Biochimica et Biophysica Acta, 1161(1), 19–27. 8380721 10.1016/0167-4838(93)90190-3

[emi413186-bib-0006] Constantino, H.R. , Brown, S.H. & Kelly, R.M. (1990) Purification and characterization of an α‐glucosidase from a hyperthermophilic archebacterium, *Pyrococcus furious*, exhibiting a temperature optimum of 105°C and 115°C. Journal of Bacteriology, 172, 3654–3660. 2163383 10.1128/jb.172.7.3654-3660.1990 PMC213339

[emi413186-bib-0007] Durbin, R. , Eddy, S.R. , Krogh, A. & Mitchison, G. (1998) Biological sequence analysis: probabilistic models of proteins and nucleic acids. Cambridge, UK: Cambridge University Press.

[emi413186-bib-0009] Ellen, A.F. , Albers, S.V. & Driessen, A.J.M. (2010) Comparative study of the extracellular proteome of *Sulfolobus* species reveals limited secretion. Extremophiles, 14, 87–98. 19957093 10.1007/s00792-009-0290-y PMC2797410

[emi413186-bib-0010] Ellen, A.F. , Albers, S.V. , Huibers, W. , Pitcher, A. , Hobel, C.F. , Schwarz, H. et al. (2009) Proteomic analysis of secreted membrane vesicles of archaeal *Sulfolobus* species reveals the presence of endosome sorting complex components. Extremophiles, 13, 67–79. 18972064 10.1007/s00792-008-0199-x

[emi413186-bib-0011] Frenkiel‐Krispin, D. , Levin‐Zaidman, S. , Shimoni, E. , Wolf, S.G. , Wachtel, E.J. , Arad, T. et al. (2001) Regulated phase transitions of bacterial chromatin: a non‐enzymatic pathway for generic DNA protection. The EMBO Journal, 20(5), 1184–1191. 11230141 10.1093/emboj/20.5.1184 PMC145506

[emi413186-bib-0012] Gindner, A. , Hausner, W. & Thomm, M. (2014) The TrmB family: a versatile group of transcriptional regulators in archaea. Extremophiles, 18(5), 925–936. 25116054 10.1007/s00792-014-0677-2 PMC4158304

[emi413186-bib-0013] Huberts, D.H. & Van der Klei, I.J. (2010) Moonlighting proteins: an intriguing mode of multitasking. Biochimica et Biophysica Acta, 1803, 520–525. 20144902 10.1016/j.bbamcr.2010.01.022

[emi413186-bib-0014] Hugouvieux‐Cotte‐Pattat, N. , Condemine, G. , Nasser, W. & Reverchon, S. (1996) Regulation of pectinolysis in *Erwinia chrysanthemi* . Annual Review of Microbiology, 50, 213–257. 10.1146/annurev.micro.50.1.213 8905080

[emi413186-bib-0015] Jeffery, C.J. (2003) Moonlighting proteins: old proteins learning new tricks. Trends in Genetics, 19(8), 415–417. 12902157 10.1016/S0168-9525(03)00167-7

[emi413186-bib-0016] Kanehisa, M. (2002) The KEGG database. Novartis Foundation Symposium, 247, 91–252. 12539951

[emi413186-bib-0017] Kengen, S.W.M. , Luesink, E.J. , Stams, A.J. & Zehnder, A.J. (1993) Purification and characterization of an extremely thermostable beta‐glucosidase from the hyperthermophilic archaeon *Pyrococcus furiosus* . The Journal of Biological Chemistry, 213, 305–312. 10.1111/j.1432-1033.1993.tb17763.x 8477701

[emi413186-bib-0018] Kim, M. , Park, S. & Lee, S.J. (2016) Global transcriptional regulator TrmB family members in prokaryotes. Journal of Microbiology, 54, 639–645. 27687225 10.1007/s12275-016-6362-7

[emi413186-bib-0019] Koerdt, A. , Gödeke, J. , Berger, J. , Thormann, K.M. & Albers, S.V. (2010) Crenarchaeal biofilm formation under extreme conditions. PLoS One, 5(11), e14104. 21124788 10.1371/journal.pone.0014104 PMC2991349

[emi413186-bib-0020] Lee, S.J. , Engelmann, A. , Horlacher, R. , Qu, Q. , Vierke, G. , Hebbeln, C. et al. (2003) TrmB, a sugar‐specific transcriptional regulator of the trehalose/maltose ABC transporter from the hyperthermophilic archaeon *Thermococcus litoralis* . The Journal of Biological Chemistry, 278(2), 983–990. 12426307 10.1074/jbc.M210236200

[emi413186-bib-0021] Lee, S.J. , Moulakakis, C. , Koning, S.M. , Hausner, W. , Thomm, M. & Boos, W. (2005) trmB, a sugar sensing regulator for ABC transporter genes in *Pyrococcus furiosus* exhibits dual promoter specifity and is controlled by different inducers. Molecular Microbiology, 57, 1797–1807. 16135241 10.1111/j.1365-2958.2005.04804.x

[emi413186-bib-0022] Lee, S.J. , Seitz, S. , Surma, M. , Hausner, W. , Thomm, M. & Boos, W. (2007) Differential signal transduction via TrmB, a sugar sensing transcriptional repressor of *Pyrococcus furiosus* . Molecular Microbiology, 64(6), 1499–1505. 17504272 10.1111/j.1365-2958.2007.05737.x

[emi413186-bib-0023] Lee, S.J. , Surma, M. , Hausner, W. , Thomm, M. & Boos, W. (2008) The role of TrmB and trmBL‐like transcriptional regulators for sugar transport and metabolism in the hyperthermophilic archaeon *Pyrococcus furiosus* . Archives of Microbiology, 190(3), 247–256. 18470695 10.1007/s00203-008-0378-2

[emi413186-bib-0024] Lee, S.J. , Surma, M. , Seitz, S. , Hausner, W. , Thomm, M. & Boos, W. (2007) Characterization of the TrmB‐like protein, PF0124, a TGM‐recognizing global transcriptional regulator of the hyperthermophilic archaeon *Pyrococcus furiosus* . Molecular Microbiology, 65(2), 305–318. 17587231 10.1111/j.1365-2958.2007.05780.x

[emi413186-bib-0025] Livak, J.K. & Schmittgen, T.D. (2001) Analysis of relative gene expression data using real‐time quantitative PCR and the 2(‐Delta Delta C(T)) method. Methods, 25(4), 402–408. 11846609 10.1006/meth.2001.1262

[emi413186-bib-0026] Marteinsson, V.T. , Birrien, J.L. & Prieur, D. (1997) In situ enrichment and isolation of thermophilic microorganisms from deep‐sea vent environments. Canadian Journal of Microbiology, 43, 694e7–694e697.

[emi413186-bib-0027] Marteinsson, V.T. , Birrien, J.L. , Reysenbach, A.L. , Vernet, M. , Marie, D. , Gambacorta, A. et al. (1999) *Thermococcus barophilus* sp. nov., a new barophilic and hyperthermophilic archaeon isolated under high hydrostatic pressure from a deep‐sea hydrothermal vent. International Journal of Bacteriology, 49(2), 351–359. 10.1099/00207713-49-2-351 10319455

[emi413186-bib-0028] Maruyama, H. , Shin, M. , Oda, T. , Matsumi, R. , Ohniwa, R.L. , Itoh, T. et al. (2011) Histone and TK0471/TrmBL2 form a novel heterogeneous genome architecture in the hyperthermophilic archaeon *Thermococcus kodakarensis* . Molecular Biology of the Cell, 22(3), 386–398. 21148291 10.1091/mbc.e10-08-0668 PMC3031468

[emi413186-bib-0029] Megaw, J. & Gilmore, B.F. (2017) Archaeal persisters: persister cell formation as a stress response in *Haloferax volcanii* . Frontiers in Microbiology, 8, 1589. 28871247 10.3389/fmicb.2017.01589 PMC5566976

[emi413186-bib-0031] Mistry, J. , Chuguransky, S. , Williams, L. , Qureshi, M. , Salazar, G.A. , Sonnhammer, E. et al. (2021) Pfam: the protein families database in 2021. Nucleic Acids Research, 49(D1), D412–D419. 33125078 10.1093/nar/gkaa913 PMC7779014

[emi413186-bib-0032] Moalic, Y. , Hartunians, J. , Dalmasso, C. , Courtine, D. , Georges, M. , Oger, P. et al. (2021) The piezo‐hyperthermophilic archaeon *Thermococcus piezophilus* regulates its energy efficiency system to cope with large hydrostatic pressure variations. Frontiers in Microbiology, 12, 730231. 34803948 10.3389/fmicb.2021.730231 PMC8595942

[emi413186-bib-0033] Murina, V.N. & Nikulin, A.D. (2011) RNA‐binding Sm‐like proteins of bacteria and archaea. Similarity and difference in structure and function. The Biochemist, 76(13), 1434–1449. 10.1134/S0006297911130050 22339597

[emi413186-bib-0051] Pérez, A., Pedrós, B., Murgui, A., Casanova, M., López‐Ribot, J.L., & Martínez, J.P. (2006) Biofilm formation by Candida albicans mutants for genes coding fungal proteins exhibiting the eight‐cysteine‐containing CFEM domain. FEMS Yeast Res, 6(7), 1074–1084. 17042757 10.1111/j.1567-1364.2006.00131.x

[emi413186-bib-0035] Schmid, G. , Mathiesen, G. , Arntzen, M.O. , Eijsink, V.G. & Thomm, M. (2013) Experimental and computational analysis of the secretome of the hyperthermophilic archaeon *Pyrococcus furiosus* . Extremophiles, 17(6), 921–930. 23979514 10.1007/s00792-013-0574-0 PMC3824201

[emi413186-bib-0036] Schmittgen, T.D. & Zakrajsek, B.A. (2000) Effect of experimental treatment on housekeeping gene expression: validation by real‐time, quantitative RT‐PCR. Journal of Biochemical and Biophysical Methods, 46(1–2), 69–81. 11086195 10.1016/s0165-022x(00)00129-9

[emi413186-bib-0037] Schneider, K.L. , Pollard, K.S. , Baertsch, R. , Pohl, A. & Lowe, T.M. (2006) The UCSC archaeal genome browser. Nucleic Acids Research, 34(Database issue), D407–D410. 16381898 10.1093/nar/gkj134 PMC1347496

[emi413186-bib-0038] Sengupta, S. , Ghosh, S. & Nagaraja, V. (2008) Moonlighting function of glutamate racemase from *mycobacterium tuberculosis*: racemization and DNA gyrase inhibition are two independent activities of the enzyme. Microbiol, 154(9), 2796–2803. 10.1099/mic.0.2008/020933-0 18757813

[emi413186-bib-0039] Soler, N. , Marguet, E. , Verbavatz, J.M. & Forterre, P. (2008) Virus‐like vesicles and extracellular DNA produced by hyperthermophilic archaea of the order Thermococcales. Research in Microbiology, 159, 390–399. 18625304 10.1016/j.resmic.2008.04.015

[emi413186-bib-0040] Stoodley, P. , Sauer, K. , Davies, D.G. & Costerton, J.W. (2002) Biofilms as complex differentiated communities. Annual Review of Microbiology, 56, 187–209. 10.1146/annurev.micro.56.012302.160705 12142477

[emi413186-bib-0041] Szklarczyk, D. , Gable, A.L. , Lyon, D. , Junge, A. , Wyder, S. , Huerta‐Cepas, J. et al. (2019) STRING v11: protein‐protein association networks with increased coverage, supporting functional discovery in genome‐wide experimental datasets. Nucleic Acids Research, 47(D1), D607–D613. 30476243 10.1093/nar/gky1131 PMC6323986

[emi413186-bib-0042] Szklarczyk, D. , Kirsch, R. , Koutrouli, M. , Nastou, K. , Mehryary, F. , Hachilif, R. et al. (2023) The STRING database in 2023: protein–protein association networks and functional enrichment analyses for any sequenced genome of interest. Nucleic Acids Research, 51(D1), D638–D646. 36370105 10.1093/nar/gkac1000 PMC9825434

[emi413186-bib-0043] Thiel, A. , Michoud, G. , Moalic, Y. , Flament, D. & Jebbar, M. (2014) Genetic manipulations of the hyperthermophilic piezophilic archaeon *Thermococcus barophilus* . Applied and Environmental Microbiology, 80(7), 2299–2306. 24487541 10.1128/AEM.00084-14 PMC3993132

[emi413186-bib-0044] Van de Werken, H.J. , Verhees, C.H. , Akerboom, J. , de Vos, W.M. & van der Oost, J. (2006) Identification of a glycolytic regulon in the archaea *Pyrococcus* and *Thermococcus* . FEMS Microbiology Letters, 260(1), 69–76. 16790020 10.1111/j.1574-6968.2006.00292.x

[emi413186-bib-0045] Vannier, P. , Marteinsson, V.T. , Fridjonsson, O.H. , Oger, P. & Jebbar, M. (2011) Complete genome sequence of the hyperthermophilic, piezophilic, heterotrophic, and carboxydotrophic archaeon *Thermococcus barophilus* MP. Journal of Bacteriology, 193(6), 1481–1482. 21217005 10.1128/JB.01490-10 PMC3067617

[emi413186-bib-0046] Vannier, P. , Michoud, G. , Oger, P. , Marteinsson, V.Þ. & Jebbar, M. (2015) Genome expression of *Thermococcus barophilus* and *Thermococcus kodakarensis* in response to different hydrostatic pressure conditions. Microbiological Research, 166(9), 717–725. 10.1016/j.resmic.2015.07.006 26239966

[emi413186-bib-0047] Waskom, M.L. (2021) seaborn: statistical data visualization. Journal of Open Source Software, 6(60), 3021.

[emi413186-bib-0048] Whittaker, C.A. & Hynes, R.O. (2002) Distribution and evolution of von Willebrand/integrin a domains: widely dispersed domains with roles in cell adhesion and elsewhere. Molecular Biology of the Cell, 13(10), 3369–3387. 12388743 10.1091/mbc.E02-05-0259 PMC129952

[emi413186-bib-0049] Zeng, X. , Birrien, J.L. , Fouquet, Y. , Cherkashov, G. , Jebbar, M. , Querellou, J. et al. (2009) *Pyrococcus* CH1, an obligate piezophilic hyperthermophile: extending the upper pressure‐temperature limits for life. The ISME Journal, 3(7), 873–876. 19295639 10.1038/ismej.2009.21

[emi413186-bib-0050] Zhao, L. , Poschmann, G. , Waldera‐Lupa, D. , Rafiee, N. , Kollmann, M. & Stühler, K. (2019) OutCyte: a novel tool for predicting unconventional protein secretion. Scientific Reports, 9(1), 19448. 31857603 10.1038/s41598-019-55351-z PMC6923414

